# Bio-Inspired Nanomembranes as Building Blocks for Nanophotonics, Plasmonics and Metamaterials

**DOI:** 10.3390/biomimetics7040222

**Published:** 2022-12-01

**Authors:** Zoran Jakšić, Marko Obradov, Olga Jakšić

**Affiliations:** Center of Microelectronic Technologies, Institute of Chemistry, Technology and Metallurgy, National Institute of the Republic of Serbia, University of Belgrade, 11000 Belgrade, Serbia

**Keywords:** nanomembranes, nano-optics, nanophotonics, metamaterials, plasmonics, photodetectors, biosensors, chemical sensors, nanoantennas, photocatalysis

## Abstract

Nanomembranes are the most widespread building block of life, as they encompass cell and organelle walls. Their synthetic counterparts can be described as freestanding or free-floating structures thinner than 100 nm, down to monatomic/monomolecular thickness and with giant lateral aspect ratios. The structural confinement to quasi-2D sheets causes a multitude of unexpected and often counterintuitive properties. This has resulted in synthetic nanomembranes transiting from a mere scientific curiosity to a position where novel applications are emerging at an ever-accelerating pace. Among wide fields where their use has proven itself most fruitful are nano-optics and nanophotonics. However, the authors are unaware of a review covering the nanomembrane use in these important fields. Here, we present an attempt to survey the state of the art of nanomembranes in nanophotonics, including photonic crystals, plasmonics, metasurfaces, and nanoantennas, with an accent on some advancements that appeared within the last few years. Unlimited by the Nature toolbox, we can utilize a practically infinite number of available materials and methods and reach numerous properties not met in biological membranes. Thus, nanomembranes in nano-optics can be described as real metastructures, exceeding the known materials and opening pathways to a wide variety of novel functionalities.

## 1. Introduction

The advent of nanotechnologies has led humanity to a silent revolution in material science and engineering. Slowly but steadily generations of researchers have been striving to make the visionary dream of the father of nanotechnology research, Richard Feynman, come true and nearing his prophetic 1959 words, “There’s plenty of room at the bottom” [1]. The possibility of engineering materials on their most basic level, starting from the rearrangement of atoms and molecules, has brought us to the material properties and abilities beyond our wildest expectations [2].

One of the paradigm-shifting advancements in the field of nanotechnologies is the ability to mimic biological structures and use and enhance their functionalities. The most basic building blocks of life as we know it are biological nanomembranes that include cell and organelle walls. They ensure mechanical protection and maintain the shape of the living cells and their organelles, including the nucleus. However, the key role belongs to the built-in parts in living membranes like ion channels, ion pumps, or nuclear pore complexes that functionalize them and enable them to fulfill their roles in biology. They appear literally everywhere, from the simplest single-cell life forms to the most complex and highly sophisticated organisms like humans and other mammals. One of the parts of the silent breakthrough occurred when advanced nanofabrication technologies enabled us to manufacture artificial composite structures in the similar dimensions range as the biological membranes. The next step was achieved when various functionalization methods of such artificial counterparts of the living membranes were introduced, enabling us to achieve at least a part of the performance of the living cells.

One of the two main directions of the related research has been directed towards fabricating replicas of the living cell walls, their model structure being the lipid bilayer. Its artificial counterparts are known as “black lipid membranes” or “painted bilayers”. A survey of synthesis methods of lipid bilayer membranes can be found in [3]. This research path also includes functionalization mechanisms mimicking those of biological membranes—for instance incorporating artificial ion channels [4], ion pumps [5], nuclear pore complexes [6,7], etc. Albeit enormous advances have been achieved in this area, they stopped short in front of the most formidable task of them all: how to create a new synthetic living cell out of the non-living building blocks—“synthetic biogenesis” [8].

Another direction of nanomembrane research is the fabrication of synthetic nanomembranes out of the building blocks not used in nature and directed to functionalities only loosely inspired by the biological ones, except at the very basic level—and often introducing completely new functionalities. To this purpose, a vast number of materials and processes have been used that are not met in biological structures. A plethora of research results have been achieved in this way: novel mechanical, electrical, chemical, biochemical, optical, plasmonic properties and many more, many of them peculiar and seemingly counterintuitive [9,10].

One could imagine a point where these two divergent branches of research meet, bringing us to a quantum leap in research as yet another paradigm shift emerges. It would lead us to integration of the living or quasi-living units with functionalities unrelated to biology and life sciences. In this way two opposites would finally unite, potentially bringing us to results surpassing even the most extreme expectations.

Among the vast fields that may at a first glance seem unrelated to biology and life sciences are nano-optics, the branch of optics dedicated to the investigation of optical phenomena at nanoscale, and the nanophotonics, the branch of nano-optics dealing with its practical applications. Nano-optics and nanophotonics have already brought us to breaking the limits that previously had been taken for granted and were almost regarded as axioms. Among those are overcoming the Abbe limit [11], a possibility to pack the light with a free-space wavelength of several hundred of nanometers into volumes of few tens of nanometers, to make ultracompact and ultrafast all-optical integrated circuits [12], to sense single molecules [13], to arbitrary tailor refractive index, reaching near-zero values [14], extremely high ones [15] and, probably the most researched of them all, negative values [16,17].

In reality a vast number of the applications of nano-optics and nanophotonics quickly appeared in biomedicine and life sciences and now are actually used in them. They range from ultrasensitive and ultra-selective biosensors [18] to various medical treatment methodologies including those aimed to cancer cells eradication, probably the most prominent ones being those based on thermoplasmonics [19]. There is an extremely large number of biomedical applications of nanophotonics (e.g., [20,21] and it appears as if a significant part of its practical uses is actually dedicated to this field. A similar situation is encountered with bio-inspired nanomembranes, which actually do have a lot in common with nano-optics/nanophotonics, since ultrathin membranes are becoming an unavoidable building block in many active and passive photonic devices (e.g., [22]).

This work is dedicated to an attempt to survey the state of the art of research in that area of research where bio-inspired nanomembranes overlap with nano-optics/nanophotonics. The review is structured as follows: first, the nanomembrane materials, structures, and general methods behind many of the current research works in nanophotonics are briefly overviewed. In the Section 3, mesoscopic nanophotonic structures—such as nanomembrane-based photonic crystals—and their applications are presented, with an obvious accent to 2D photonic bandgap structures. The Section 4 handles the subwavelength conductor-dielectric nanocomposites belonging to the wide field of plasmonics, again with choice application. This logically leads to the Section 5 dedicated to another kind of subwavelength nanocomposites, metamaterials (more precisely, their 2D version, metasurfaces), that offers even larger level of tailorability of electromagnetic fields. The Section 6 offers an overview of planar nanoantennas built on nanomembranes. The Section 7 presents the applications in general nanophotonics not covered by the prior sections, while the Section 8 draws general conclusions and outlines potential strategies for future works.

## 2. Materials and Methods

The basic building block of all quasi-2D structures considered throughout this work are synthetic nanomembranes. In this work we use the definition of a nanomembrane as an inorganic, organic, or hybrid quasi-2D artificial freestanding (unbacked) structure with a thickness typically below 100 nm. The bottom limit of thickness is that of a single atom or a single molecule. At that, nanomembranes will have a large thickness-to-lateral size aspect ratio of at least several orders of magnitude [9].

As far as the constitutive materials of the nanomembranes go, some of them are summarized in Table 1. This is only a small part of materials that have been used to create unbacked sheets with a thickness typically below 100 nm.

We analyze constitutive materials strictly from the optical/electromagnetic point of view. This basically means that we only need knowledge about the values of their complex relative dielectric permittivity ε = ε_1_ + *i*·ε_2_, complex relative magnetic permeability μ = μ_1_ + *i*·μ_2_, and complex refractive index *n* = *n*_1_ + *i*·*n*_2_, where $n=\sqrt{\mu \;\varepsilon }$. From that point of view, only three main groups of materials are used: lossless dielectrics (the imaginary part of the refractive index is 0 and the real part is constant throughout the spectral range observed), semiconductors (used here as lossy dielectrics, meaning that the imaginary part of the refractive index is non-zero, however since generally the thickness of the building blocks of nanocomposites constituting a nanomembrane is in the nanometer range, the absorption losses caused by the existence of the imaginary component will still often be negligible), and free electron conductors (both the real and the imaginary part of the refractive index are non-negligible and absorptive losses are high).

The electron conductors, which are also denoted as plasmonic materials, must be lossy to preserve the causality principle. An often utilized form for the effective ε and μ is the lossy Drude model [38] according to which polarization is given by permittivity in the form

(1) $$\varepsilon_{eff}\left(\omega \right)=1\;\frac{\omega_{pe}^{2}}{\omega \left(\omega +i\Gamma_{e}\right)}$$

while, in an equivalent manner, magnetization can be presented through permeability as follows

(2) $$\mu_{eff}\left(\omega \right)=1\;\frac{\omega_{pm}^{2}}{\omega \left(\omega +i\Gamma_{m}\right)}$$

where plasma frequency ω*_p_* and dumping constant Γ are assumed to be equal for both electric and magnetic behavior

(3) $$\omega_{pe}=\omega_{pm}=\omega_{p},\;\Gamma_{pe}=\Gamma_{pm}=\Gamma_{p}$$

This text is mostly dedicated to nanomembranes functionalized by specific types of electromagnetic scatterers for the optical wavelength range. As far as mesoscopic or subwavelength scatterers are generally concerned, regardless of if they are utilized within the context of nanomembranes, they may be 1D, 2D, or 3D spatially arranged.

In this work, we consider mostly 2D layouts. The reason behind that is that if we try to utilize 3D arrangements of subwavelength scatterers as building blocks for nanomembrane functionalization, we will find that usually these arrangements have to be excessively thick compared to typical nanomembrane dimensions. Since, as a rule, the scatterer arrangements need to have at least several structural periods to achieve their functionality, this means that they tend to exceed 100 nm, the value usually posed as the upper limit of thickness to nanomembranes. This issue is elaborated in more detail in Section 3 dedicated to photonic crystal nanomembranes. There are notable exceptions, however, which include subwavelength structures such as plasmonic crystals and metamaterials, where 1D geometries may be used to form optically functionalized nanomembranes, even the multilayered (laminated) ones. Generally, 3D geometries are never used within this context. Another reason why they are avoided is of technological nature; most of micro and nanofabrication methods are tailored to furnish planar structures, which means that 2D fabrication procedures and protocols are best defined and most mature. In general, designing and tailoring nanoscale 3D structures is still simply too complex, albeit even this could be partly overcome, for instance by self-assembly technologies.

**Figure 1:**
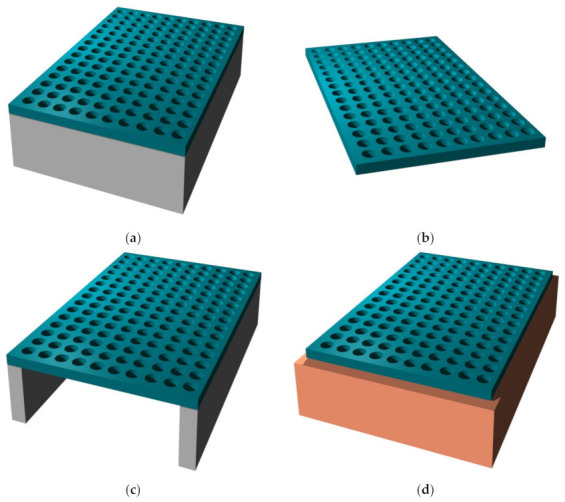
Top-down method for nanomembrane fabrication by sacrificial etching. (**a**)Starting structure where the membrane material is deposited to a sacrificial substrate. The membrane is then functionalized by scatterers (in this case, a square lattice of nanoapertures; this step may be done, e.g., by focused ion beam etching and is not shown in the figure); (**b**) the sacrificial support is completely removed by wet etching and the membrane is left free-floating in the solution (in reality the membrane will not be perfectly flat and its surface will freely undulate in the solution); (**c**) one possible way to deal with the membrane is to transfer it to a support. For the sake of clarity, the front part of the support is not shown; the support can be fabricated immediately after the step (**a**) if only a part of the sacrificial support had been removed, using, e.g., a lithographic mask and bulk micromachining (anisotropic wet etching); (**d**) another alternative, where the free-floating membrane has been transferred to another substrate, different from the original one.

Among multitudinous methods to functionalize nanomembranes and generally quasi-2D structures by nano-scatterers for optical purposes (see, e.g., [9] or [10]), we opt to present in Figure 1 what is probably the most often used top-down method: the sacrificial etching. This figure is a general illustration of the methodology predominantly used to fabricate freestanding nanomembranes. Naturally, the particular etching solution will depend on the material used for the sacrificial substrate. For instance, if the sacrificial material is silicon (as it often is), and one intends to keep its rim as the membrane holder, then one will wish to use anisotropic etchant solutions such as those containing potassium hydroxide (KOH), ethylenediamine pyrocatechol (EDP), tetramethylammonium hydroxide (TMAH) or hydrazine, combined with a photomask on the bottom part of the sacrificial substrate, opposite to the membrane. If no rim is needed, one can also use isotropic wet etching, typically done in HNA (mixture of hydrofluoric, nitric and acetic acid). All of these wet etching methods belong to the fundamental cornerstones of bulk micromachining [39]. An in-depth view of technological procedures for nanomembrane, listing both the quoted and many other approaches to fabrication, together with the pertinent details, can be found in [9].

**Figure 2:**
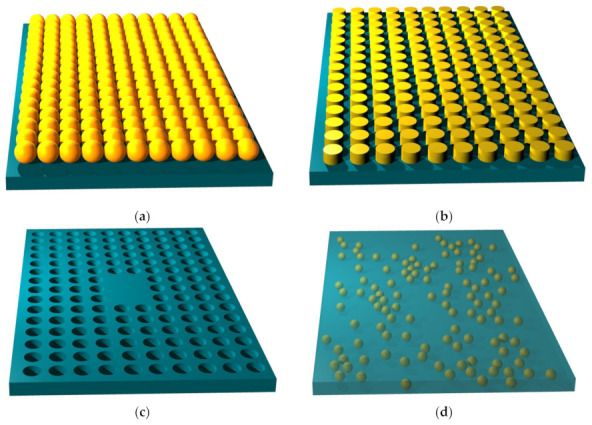
Functionalization of nanomembrane by scatterers. (**a**) self-assembled metal nanospheres fixed to the nanomembrane surface (bottom-up approach); (**b**) deposition of cylindrical metal (gold) scatterers on the nanomembrane (top-down); (**c**) square lattice of circular apertures; middle six apertures are intentionally left out, making a defect in the lattice (an optical nanocavity in 2D); (**d**) randomly scattered nanospheres within the nanomembrane host material (the nanofiller approach).

The scatterers may be deposited on the nanomembrane substrate (additive method that makes protrusions on the surface), or they may be etched in the membrane, making either indentations (pits) or full apertures which go throughout the membrane (the subtractive method). Self-assembly (bottom-up) methods may be also used for functionalization. Figure 2 illustrates some of these possibilities. The reader should observe that all structure except the one shown in Figure 2c contain metal scatterers. This is of crucial importance for their function, since metals are the main plasmonic materials and thus ensure most of the plasmonic and metamaterial functionalities, as explained in detail in the further text. Obviously, a vast number of other fabrication and functionalization methods exist. For a review of some of them, the reader may wish to consult [40].

Lamination or multilayering is a functionalization method used in 1D plasmonics and 1D and 2D metamaterials. An important functionalization method by lamination is used with 2D materials like graphene or borophene, etc., and is denoted as a twisted bilayer formation. Within it, a single-crystalline monolayer is deposited on another monolayer, but rotated in-plane at a certain angle to obtain a twisted bilayer. The resulting structure forms a moiré pattern whose structure depends on the twisting angle. Under a certain angle (the “magic angle”) the twisted bilayer arrives at completely different electronic and optical behavior in comparison to the same structure and material without twisting [32,41]. This important new behavior is investigated within the new research fields of twistronics or moiré physics [42,43].

A combination of subtractive functionalization and lamination is fundamental for some very important applications as some highly significant and practical novel optical properties have been thus obtained [44]. This will be duly considered in Section 5 on negative refractive index metamaterials.

**Figure 3:**
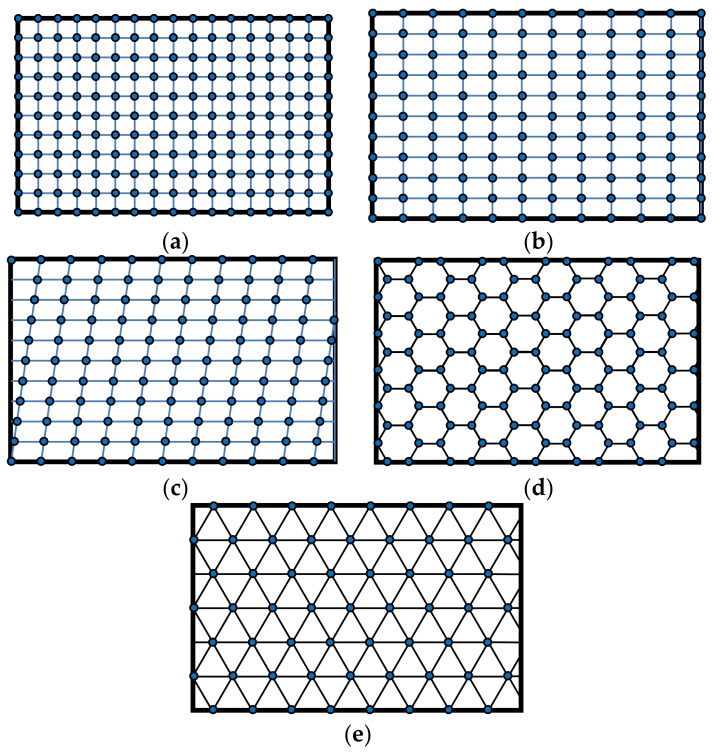
Five basic types of 2D Bravais spatial lattices of the electromagnetic scatterers integrated with nanomembranes: (**a**) square lattice (belongs to tetragonal group); (**b**) rectangular (orthorhombic group); (**c**) oblique (monoclinic group); (**d**) hexagonal (hexagonal group); (**e**) triangular–centered hexagonal (hexagonal group).

**Figure 4:**
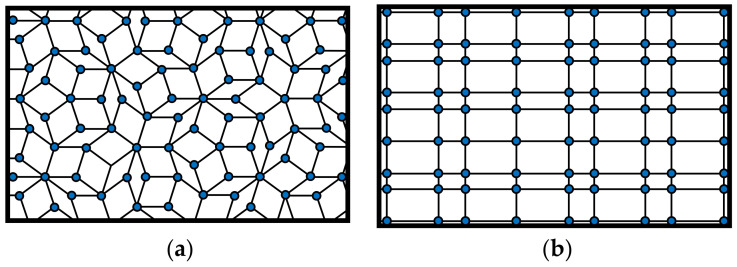
Some quasiperiodic/aperiodic 2D spatial lattices of the electromagnetic scatterers. (**a**) Penrose quasicrystal (aperiodic lattice); (**b**) 2D square Fibonacci sequence lattice.

As far as the geometrical distribution of scatterers related to a nanomembrane-based photonic element are concerned, it may be periodic (alternating with a given spatial period, i.e., having their unit cell and lattice constant fully defined), Figure 3. It may also be quasiperiodic/aperiodic, Figure 4, or random. Distribution examples also include quasicrystals (aperiodic structures) and graded ones.

A question may be posed how one calculates the electromagnetic behavior of the ordered scatterers in 2D lattices, and the general reply is by using numerical simulation because of the relative complexity of the systems that are calculated [45]. Modeling nanomembranes for nanophotonics, especially those containing plasmonic materials, can pose formidable problems because of the appearance of field hotspots that require extremely fine meshing and thus very long computation times. Among most often used approaches is the application of Finite Elements Method to a single unit cell of the lattice, together with periodic boundary conditions to ensure that the results are valid throughout the whole structure. This is far from being the only simulation method used to calculate scattering parameters (transmissivity and reflectivity) and generally optical parameters and behavior of the structure. An even larger numerical problem is met with aperiodic/quasiperiodic structures (Figure 4) where no periodic boundary conditions exist [46].

Importantly, nanomembranes offer a number of additional advantages for nanophotonic applications, unrelated to their optical behavior but still very useful for their practical application in photonics. These include their relatively facile transferability to any arbitrary substrates and supports/handlers [47,48], their self-healing properties [49] where the nanomembrane spontaneously repairs itself, most usually based on cross-linking within the nanomembrane structure, their extreme stretchability, flexibility and foldability [50], the possibility of 3D sculpting [51,52], antifouling properties [53], etc. Most of them are used to impart multifunctionality to nanophotonic elements and devices.

## 3. Photonic Crystals Based on Nanomembranes

### 3.1. Introductory Remarks

Photonic bandgap (PBG) materials, more commonly known as photonic crystals, represent a class of synthetic composite optical materials in which their complex refractive index is spatially varied [54,55,56]. Most often it is alternating periodically and with large enough contrast between the segments with a low and a high real part of the complex refractive index (typical contrasts range between 1.5 and 2). The spatial periodicity of the photonic crystals for the visible wavelengths is of the order of few hundred of nanometers, the size comparable with a quarter of the operating wavelength (mesoscopic structures). The spatial variations can be along one, two, or three dimensions. Photonic crystal properties can even be variable with time (the co-called spatiotemporal photonic crystals [57]).

**Figure 5:**
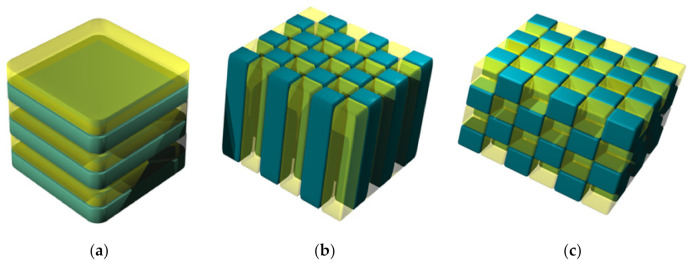
Basic geometries of periodic photonic crystals: (**a**) 1D structure (laminar), periodicity goes along one direction; (**b**) 2D structure–in-plane periodicity; (**c**) full 3D periodicity. Dark parts denote high refractive index regions, while light parts are regions with lower index.

Typically, dielectric materials are used to build photonic crystals, although metal-dielectric combinations are also met. Figure 5 illustrates the 1D, 2D, and 3D geometry of photonic crystals. The pioneer of photonic crystals rejected the opinion that 1D structures are actually photonic crystals and opined that they do not have a photonic bandgap, but actually only a bandstop, and that they should be described simply as Bragg mirrors or dielectric mirrors. The question has remained debatable since, but it appears that the majority of the scientific community nevertheless adopted the term “1D photonic crystal” in spite of dissenting opinions.

Photonic crystals may be regarded as a generalization of common 1D multilayer dielectric mirrors [58] since the higher dimensionality of photonic crystals ensures either confinement of electromagnetic waves within a plane as in 2D structures or in full 3D, making them behave as real omnidirectional mirrors. In all cases (1D, 2D, or 3D), a range of frequencies appears where the propagation of light is prevented by multiple internal reflections within the structure. This is a behavior similar to what the semiconductor crystal lattice does with charge carriers where the lattice also creates a band of energies and the charge carrier propagation modes within that range are prohibited. Since that band is the well-known energy bandgap of semiconductors, its equivalent in photonic crystals has been denoted as the photonic bandgap (PBG).

There are numerous similarities and analogies between the semiconductor crystals and photonic crystals. Among other things, both exhibit defect modes, can have acceptor and donor dopants (in PBG structures these are the building blocks with either different size or different refractive index than the rest of the structure, so that they also have their own kind of “donors” and “acceptors”), exhibit surface states and surface waves, etc. Their crystal structure has its unit cells with the order of magnitude of the operating wavelength.

The methods of calculation of the relation between the PBG and the refractive index values are numerous and include both analytic and numerical ones. While the simple transfer matrix method [59] can be used for 1D photonic crystals, 2D and 3D structures are usually calculated by commercial software packages based on, e.g., Finite Element Method, Finite Difference Time Domain Method, boundary elements method, etc. (a more in-depth approach can be found in [60].

### 3.2. Photonic Crystal Nanomembranes

Nanomembrane-based photonic crystals are typically two-dimensional (Figure 5b), since their thickness along the direction normal to the surface can be decreased to the nanoscale range, i.e., below 100 nm, while keeping the functionality. The thinnest 2D PBG materials for the visible optical range were experimentally demonstrated in an atomically thick (i.e., at the angstrom thickness limit) tungsten disulfide membrane patterned as a photonic crystal structure [61]. The 1D and 3D structures are not usable within the context of nanomembranes because both have to be periodic along the direction normal to the surface of the nanomembrane. Since a single period of a PBG structure is about a quarter of the operating wavelength, the very condition of the existence of the PBG requires structures, the pre-adopted maximum thickness limit of a nanomembrane of 100 nm will be exceeded even for a single period. The same is valid for both horizontal axes in the 3D case, as well as for the lateral dimensions in 2D photonic crystals, while in the 1D case one could imagine a multilayer structure whose one or even two lateral dimensions added together would be below 100 nm. However, the edge effects in such structures would be so large that the appearance of a bandstop range would be hardly feasible, while the very form of the structure would make it hard to utilize it practically. On the other hand, such a structure would be more convenient for the support of surface waves and for obtaining surface states, especially for the case of metal-dielectric photonic crystals [62,63].

From the point of view of practical fabrication, 2D PBG structures appear to be the most convenient, since they ensure most of the functionalities of full 3D structures, while at the same time requiring only the use of planar technologies. Although they exhibit a full photonic bandgap only in their plane, their out-of-plane leaky waves are mostly suppressed by total internal reflection at the top and bottom interface between their high refractive index host material and the surrounding air. The result is a wave confinement that is worse than in real 3D structures with some radiation losses being inevitable, but technologically their fabrication is much simpler, so this represents an acceptable tradeoff. This is the reason why they are vastly more used in practical photonics than 3D photonic crystals.

### 3.3. Some Practical Applications

**Figure 6:**
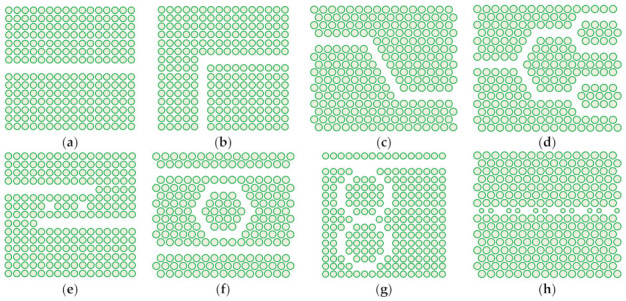
Some passive waveguide-based components in 2D photonic crystal nanomembranes, top view. (**a**) Line defect waveguide; (**b**) 90° channel waveguide in rectangular lattice; (**c**) 60° channel waveguide in triangular lattice; (**d**) two-stage Y-splitter; (**e**) cavity-based wavelength division multiplexer; (**f**) ring resonator with single loop in triangular lattice; (**g**) ring resonator in rectangular lattice with two loops in vertical layout; (**h**) coupled-resonator optical waveguide in triangular lattice.

Nanomembrane-based 2D photonic crystals are usable in a large number of practical applications. Among these are passive ultracompact waveguide components [64], like channel waveguides used for mostly lossless beam bending at distances comparable to the operating wavelength, i.e., vastly smaller than in conventional photonics, other kinds of optical waveguide structures with micro dimensions, various kinds of optical filters, beam splitters, multiplexers/demultiplexers, various optical couplers [65], different types or resonators electrically, mechanically, or optically tunable in real time, coupled resonator optical waveguide (CROW) [66] that may use defect cavities in the photonic crystal, dielectric microdisks, microrings or microcavities, polarization converters, passive optical limiters [67], on-chip optical true time delay lines [68], etc. Figure 6 shows top views of some examples of passive waveguide-based components in 2D photonic crystal nanomembranes.

Active PBG waveguide components include various advanced light sources like ultralow threshold lasers, microlasers, VCSELs (vertical-cavity surface-emitting lasers) [69], ultrahigh efficiency LEDs, but also nonlinear frequency converters, nonlinear optical switches, etc. The use of gain media as the constitutive parts of ultrathin PBG emitters with emission control implemented through an increase of the light extraction efficiency provided another boost to this branch of applications. PBG structures are also used to enhance photocatalytic processes [70].

Photonic crystals are also used a lot in conjunction with Fano-resonance enhancement. Fano resonance in nanophotonics generally is a resonant electromagnetic effect that occurs when a discrete quantum state couples with a continuum band of states through interference [71], resulting in the characteristic asymmetric line shape. This phenomenon is common in a large number of other branches of physics as well. In general nano-optics, the sharp asymmetric resonant shape is observed in absorption, transmission, and other spectral dispersions [71].

Fano resonance in 2D photonic crystals has been a popular topic of research [72]. There are different Fano-resonance based 2D photonic crystals applications [73], most notably slow light devices for optical waveguide delay lines and with large dispersion enhancement [74], ultra-broadband reflectors, optical filters, nonlinear devices, light emitters and optical detectors, and various cavity-enhanced devices.

A large group of devices enabled by ultrathin photonic structures are various photodetectors, mostly those of intrinsic photonic type where the PBG width can be tailored to coincide with the bandgap of the semiconductor. Most notably, they include resonant cavity enhanced (RCE) and photonic crystal enhanced (PCE) devices, but also different types of solar cells.

The inventor of photonic crystals himself, Eli Yablonovitch, came up with the idea of photonic crystals while investigating the conditions of light trapping within the photodetector active area. He was analyzing ways to push the detector quantum efficiency closer to the fundamental performance limits, also known as the conventional limit, the ray-optics limit, the ergodic light trapping limit or the Lambertian limit. The idea was to enclose the photodetector within a cavity with omnidirectional reflection (full 3D). Such optical path enhancement was the actual goal of his seminal paper that introduced photonic crystals to the world [54].

There is a body of literature dedicated to various methods of photodetector performance enhancement. An attempt to classify and systematize in detail different approaches, generally including the use of photonic crystals and nanophotonics, can be found in [75].

Photon management in a detector can be done by maximizing light concentration within the active region (which may be done through refractive and reflective concentrators, i.e., lenses or mirrors, but also diffractive structures like diffractive optical elements and holographic optical elements and finally through plasmonic light localization). The second approach is to use antireflection structures and includes interference multilayers, graded index dielectric films, diffractive structures, biomimetic structures like biomimetic moth-eye elements, random surface corrugations, subsurface or topside scatterers which in case of plasmonic nanoparticles or gratings enhancers additionally cause extreme light localizations, nanoantennas, and metamaterials (including superabsorbers). The third approach is to increase the optical path within the active region, and it includes the use of reflective structures such as backside mirrors, total internal reflection structures, radiative shields, resonant cavity enhancement (RCE), and photonic crystal enhancement (PCE).

Pan et al. designed and fabricated photodetectors based on roughened (surface-sculpted) silicon nanomembranes [76]. They demonstated significant dark current suppressions due to surface depletion and Schottky barrier modulations. A vast number of other works dedicated to this area of research have been published until now.

Besides increasing the quantum efficiency enhancement of photodetectors and being building blocks for various types of resonant cavities including tailorable ones, photonic crystal nanomembranes serve as a basis for the detectors themselves as their function-enabling part. Moein et al. described a photodetector with an optically thin broadband graphene-monolayer on silicon nitride nanomembrane [77]. In their recent book chapter, Kim et al. described high-performance flexible photodetectors based on silicon, germanium, and III–V compound semiconductor nanomembranes [78]. Strain-engineered germanium-nanomembranes can be tailored to convert the indirect bandgap of Ge to a direct one and be utilized for different purposes, among others to make use of nanomembrane transferability to make germanium on silicon photodetectors [79].

Obviously, depending on the constitutive materials, the optical properties of PBG materials may be tunable by different external stimuli. For instance, as seen in the previous paragraph, the presence of external mechanical influences such as pressure, strain, and flexure will modify the properties of photonic crystals. Temperature changes or heat gradients may also influence their optical behavior, as well as absorption or adsorption of chemical or biochemical analytes. This is of importance for multitudinous sensing applications of photonic crystals.

A prominent place among them belongs to ultrasensitive biological sensing of disease biomarkers, antibiotics and similar analytes [80]. Some of these devices are based on electrochemiluminescence. The photonic crystal nanomembrane is typically used for signal amplification utilizing various strategies, most often light scattering. Xiao-Yan Wang et al. [18] used silicon dioxide photonic crystal nanomembranes as the electrochemiluminescent electrodes to selectively detect SFTSV (Severe Fever with Thrombocytopenia Syndrome Virus, also known as Huaiyangshan Banyangvirus or Dabie bandavirus). Using the scheme they proposed, the team achieved a 7-fold increase of the electrochemiluminescent intensity. The same principal author with a different team used electrochemiluminescence with their gold-filled polystyrene nanomembranes to detect tetracycline antibiotic [81]. Chemical and biological sensing using photonic crystals was considered in [82]. PBG structures containing hydrogel as sensitive material responding to pH factor, humidity and temperature changes, and chemical and biological analytes were described in [83].

Mechanic tunability of optical characteristics of photonic crystals is often used in mechanical sensing, as well as in cavity-based nanoresonator fabrication. It is also used for mechanical tuning of characteristics of different membrane-based optoelectronic devices. Nanomembrane-based 2D photonic crystals are typically both stretchable and flexible. This enables altering of their mechanical and structural properties. In this manner their optical properties are modified proportionally to the applied mechanical force.

Manjeshwar et al. [84] manufactured mechanical resonators out of 100 nm-thin freestanding GaAs nanomembranes structured as 2D photonic crystals. The resonators were intended for optomechanical microcavities on chip and for multi-element cavity optomechanical devices. Lu et al. [85] fabricated photonic crystal nanocavities from periodically stacked 1D nanorods embedded in a polydimethylsiloxane membrane. Their structure served as a sensor for strain analysis, since it can recognize different planar strains, including their type, direction, and amplitude. Chen et al. [86] showed through finite element simulations that strain in photonic crystals can induce photonic topological insulator states, thus pointing towards possible future uses in in quantum computing. Zhang et al. reviewed various applications of mechanochromic photonic crystals [87]. They also considered biomimetic inspiration for tuning of structural coloring of such nanophotonic composites. They observed applicability of mechanochromic PBG structures in such diverse fields as civil engineering (visual observation of defects and structural damages through strain-induced coloring), household appliances, biomedical sensors, fingerprint sensors, and multitudinous applications where a color-changing miniature strain sensor can be of use. Earlier, Rindorf and Bang [88] investigated photonic crystal fiber grating sensors in various sensing application including temperature measurement, refractometry, biological sensing, and strain measurement. Mechanically reconfigurable membrane-based photonic crystals based on shape memory copolymers were also proposed [89]. Due to their use of photonic crystals and the choice of the materials, their complete programming and recovery processes are done at room temperature, contrary to the traditional shape memory materials which are highly thermoresponsive and require the application of heat.

There are numerous other applications that make use of stretchability and flexibility of photonic crystal membranes. Many of them draw their inspiration from nature.

## 4. Nanomembrane Plasmonics

### 4.1. Introductory Remarks

Plasmonics investigates the electromagnetic behavior of nanocomposites containing electric conductors whose conductivity is based on free electron plasma (thus the names plasmonic materials) combined with low-loss or lossless dielectrics [90,91,92]. Among most often used plasmonic materials are noble metals such as gold and silver which are exceedingly good conductors. Other metals are also used, most notably aluminum, copper, chromium, etc. Among the non-metallic free electron conductors are transparent conductive oxides such as tin oxide, indium oxide, zinc oxide, and the very often used indium tin oxide. Other materials include highly doped semiconductors, different metal alloys, different 2D materials like graphene, 2D transition metal carbides (MXenes), etc. [93,94]. Nanoparticles (NP) are most often used in plasmonic nanocomposites as plasmonic material fillers within a dielectric host [95]. They typically consist of a good metal (most often gold), although most of the above mentioned alternative plasmonic materials can be used as well. A host of different NP geometrical forms are available (the popular “nanoparticle zoo”) [96], the simplest shape being spherical.

At the interface between a material with relative dielectric permittivity ε > 0 and a conductor with free electrons whose ε < 0 an incident electromagnetic wave in the optical range can under certain conditions resonantly couple with free electron plasma in the conductor and form a combined wave consisting of a p-polarized electromagnetic wave (polariton) and the mentioned free electron plasma oscillation (plasmon). This collective oscillation is known as surface plasmon polariton (SPP). An SPP will propagate along the interface between the epsilon positive and epsilon negative (plasmonic) material. Perpendicularly to the surface this collective wave will decrease exponentially in both directions, its maximum being at the interface, i.e., it will be evanescent. SPP waves have much larger wavevector than the propagating electromagnetic wave at the same frequency, which means they need a coupling structure that will impart them an additional wave vector necessary for their existence. Such coupling structures may be, e.g., prisms (Kretschmann, Otto), but also various diffractive structures like surface reliefs, gratings, apertures, or protrusions. For the short range surface plasmon polaritons the propagation length is of the order of micrometers on bulk substrates, and theoretically exceeds a few hundred micrometer on 100 nm thick atomically smooth silver film [97]. However, this is not valid for the long-range surface plasmon polaritons.

### 4.2. Long Range Surface Plasmon Polaritons

**Figure 7:**
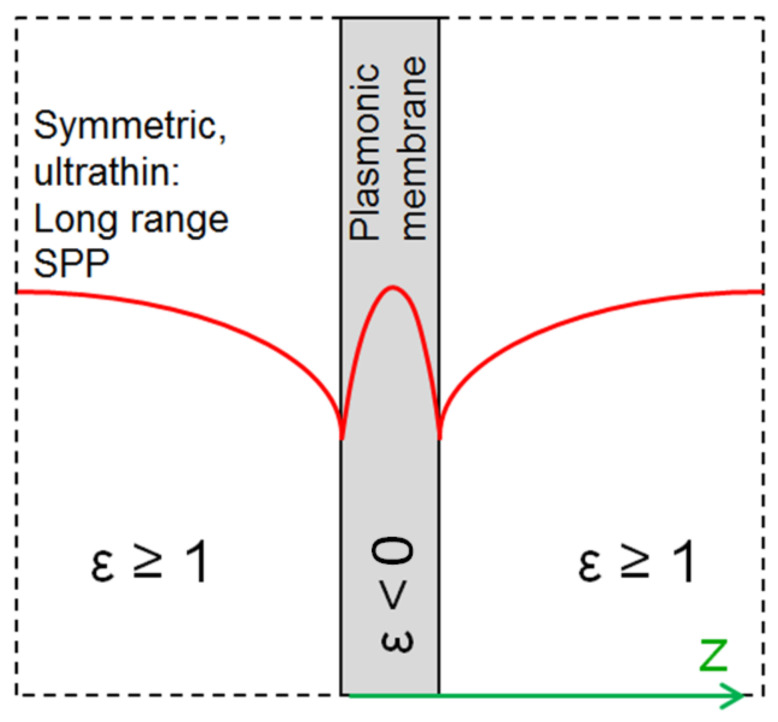
Illustration of the forming of an LRSPP supermode by coupling two surface modes on the surfaces of a plasmonic nanomembrane.

In case of the long range surface plasmon polaritons (LRSPPs), supermodes formed at ultrathin plasmonic material sheets (thickness of the order of tens of nanometers), surrounded by a dielectric with the same permittivity from both sides (electromagnetically symmetric structures), the propagation distances are much larger, of the order of centimeters [98]. The reason for such unusually large propagation lengths of the LRSPPs is that long range SPPs are less confined to the metal part and thus a larger part of their evanescent field propagates through the lossless dielectric of their ambient, meaning that the absorption losses will be proportionally lower and, consequentially, the propagation paths longer [99]. Thus on a freestanding conductive membrane surrounded by air LRSPPs will be formed; a nanomembrane is an almost ideal platform for LRSPPs [100]. Figure 7 shows the forming of a long-range surface plasmon polariton on a plasmonic nanomembrane through coupling of single modes on each side of the membrane. If one starts from a thick enough conductive slab, surface plasmon polaritons will form at each of its surfaces and will be independent on each other. With a decreasing slab thickness, the two SPPs will begin to couple. With a sufficiently thin plasmonic sheet, they will merge into a single wave–a long range surface plasmon polariton.

Alternatively, one could make a nanomembrane with ultrathin metal layer surrounded by both sides with solid dielectric. Such an alternative symmetric ambient for LRSPP waves could consist of any lossless dielectric usable for fabricating a freestanding nanomembrane. The only important optical parameter of the dielectric is its refractive index. The most important non-optical parameter is its mechanical strength, i.e., its ability to make a stable and sturdy freestanding structure with a thickness below 100 nm.

The combination of plasmonics with biomimetic nanomembranes comes to mind almost naturally when one remembers that both the evanescent field of surface plasmon polaritons and nanomembranes are limited to nanoscale thickness of a similar value, while their lateral spread is vastly larger. Freestanding nanomembranes readily reach lateral dimensions of tens of millimeters, even tens of centimeters. In their *Science* article, Jung et al. reported stretchable conductive nanomembranes with a strain up to 1000%, synthesized in 30 cm diameter container [101].

### 4.3. Subwavelength Plasmonic Crystals

Subwavelength plasmonic crystals (SPC) are nanocomposites consisting of two materials, one of which has to exhibit negative relative dielectric permittivity, while the other should have positive permittivity (most often they Drude-type conductor and lossless dielectric). Their geometry is very similar to that of photonic crystals, as shown in Figure 5. There is one significant difference, though: the dimensions of their constitutive blocks must be subwavelength.

It is relatively easy to produce 1D and 2D SPC in the form of nanomembranes using planar technologies. This is even valid for 1D laminar structures, since a stack of several layer pairs with deeply subwavelength thickness still can satisfy the dimensional requirements and can be produced by, e.g., thin-film deposition methods or by self-assembly. Besides the laminar ones, another kind of SPCs can be made by embedding plasmonic nanoparticles in a dielectric host or depositing them on its surface. Two-dimensional SPC are often made simply as arrays of apertures in a plasmonic material. The spatial arrangement of the holes may be one of the five regular ones, as shown in Figure 3, or it may be quasiperiodic/aperiodic (Figure 4) or random. The first paper that mentioned subwavelength plasmonic crystals was written by Shvets and Urzhumov [102], and 2D quasiperiodic plasmonic crystals were described in [103].

The properties of SPCs like their physical size and the geometrical arrangement, together with the complex refractive index of the constitutive materials of these nanocomposites, play the crucial role in defining their optical parameters. Usually, the surfaces of a SPC are smooth at nanoscale, often even atomically smooth, so that in principle, the morphology plays far lesser role. Rough or sculpted surfaces would increase losses by coupling evanescent SPP waves into propagating free space modes, letting them “leak” into the ambient.

The extraordinary optical transmission (EOT) structures can be also classified as subwavelength plasmonic crystals. Discovered by Ebbesen et al. [104], they represent an array of subwavelength apertures in opaque plasmonic material. According to the classical theory of Bethe, they should not be able to transmit an optical beam with a wavelength larger than the aperture diameter. Counterintuitively, they have a strong and wide transmission peak determined by the plasmonic mechanisms. EOT structures can also be considered as a plasmonic metamaterial of the single-layer fishnet type. Later it was found that the EOT effect can also occur in materials with indentations and surface reliefs, i.e., completely without apertures [105].

### 4.4. Applications of Nanomembrane-Based Plasmonics

In recent years, the field of membrane plasmonics literally exploded with a vast number of novel applications. One of the most important properties of plasmonics is its strong light localization, which has brought to numerous practical applications. The use of nanoparticle-based plasmonics has been vastly more spread than the use of plasmonics of ultrathin layers. However, this has changed drastically with the arrival of the functionalization of non-plasmonic nanomembranes (even the biological ones) with plasmonic nanoparticles, albeit even today the particle plasmonics strongly exceed membrane plasmonics.

The application number one of both nanomembrane plasmonics and general plasmonics is refractometric adsorption-based chemical and biological sensing [106]. Such sensors utilize surface plasmon resonance to offer extremely fast detection and label-free operation. They are able to reach extreme sensitivities, down to single-molecule sensing [107,108]. Besides being the most widespread, this is probably the most accomplished and mature application of plasmonic nanomembranes and plasmonics in general. It is also one of the liveliest ones, new research being intensively performed and novel discoveries appearing on a daily basis.

Bio-inspired applications using plasmonic nanomembranes are described in a number of publications, some of which we present here. The paper [109] describes a possibility to significantly enhance the performance of plasmonic nanopore chemical and biological sensors, thus achieving single-molecule sensitivity. To this purpose, a combination of plasmonic nanocavities, nanoantennas, and nanopores is investigated. Further advantageous multifunctionalities are obtained by combining these structures with lipid bilayer membranes in a biomimetic fashion, as well as by combining them with 2D materials like graphene or MXenes.

A direct combination of lipid bilayer membranes with plasmonic nanoparticles to achieve sensing with single-particle resolution is described in [110]. This platform also opens a path to applying different kinds of external stimuli to biological systems, including optical, thermal, or mechanical. A whole new area has appeared in biomimetic plasmonics dedicated to interfacing lipid bilayers modified by plasmonic nanoparticles with biological systems (including living organisms), and also with molecular circuits [111]. Another novel concept connected with this field is biocomputing with plasmonic nanostructures integrated with lipid bilayers [112].

**Figure 8:**
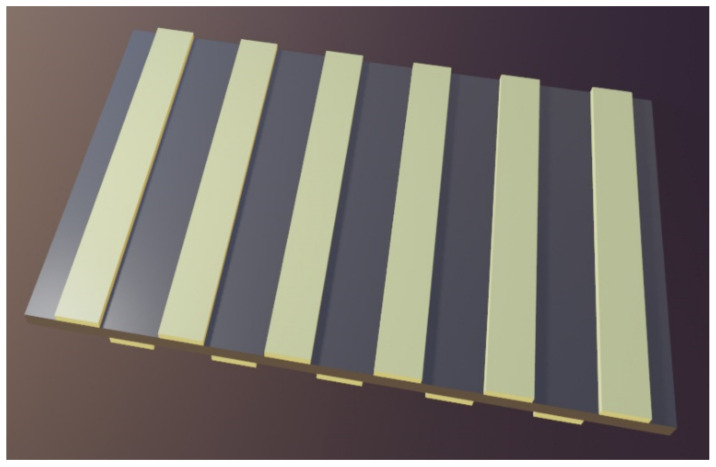
Freestanding dual plasmonic waveguide with two arrays of golden nanostrips, one on the top surface of the dielectric (silica) nanomembrane and the other on the bottom, laterally shifted to each other. The thickness of gold nanostrips is 10 nm, their width 450 nm, while the thickness of freestanding silica is 20 nm and the lateral shift between two successive nanostrips is 550 nm.

Plasmonic waveguides [113] and plasmonic circuits [114] that incorporate them represent another important application area. This kind of waveguides supports propagation of surface plasmon polaritons, thus enabling subwavelength localization of light far beyond the diffraction limit. Most of the current research of plasmonic waveguides is directed either towards making plasmonic circuits, refractometric sensors, passive devices like plasmonic couplers, resonators, interferometers, various optical modulators, switches, gates, or active devices such as photon emitters. Typically the plasmonic waveguides will consist of a plasmonic nanowire ridge on a dielectric or semiconductor substrate [114], although many other structures are utilized as well, e.g., 1D plasmonic nanoparticle arrays, electrically conductive grooves, slits, and sharp metal wedges. Recently a self-supported dual plasmonic waveguide was presented [115], Figure 8, which was designed and fabricated to use electromagnetically induced transparency and slow light in order to achieve extremely narrow transmission peaks (in experiments: about 0.14 nm) and at the same to reach very low losses (transmission higher than 92%), all of which is convenient for high performance nanophotonic devices.

A somewhat unexpected application field of plasmonic membranes is the separation technology. Thermoplasmonics (heating of a medium ionic lattice due to plasmonic localization of electromagnetic field) [116] can promote transmembrane flow by rising the temperature (the process is known as membrane distillation) where plasmonic nanoparticles integrated with the nanomembrane act as “nanoheaters”. The result is that membrane thermoplasmonics enhance filtration, e.g., solvent-resistant nanofiltration [117]. A similar process is used in nanomembrane-based water desalination [118].

Plasmonic membranes are also used in nanolithography [119], solar cell enhancement (photovoltaics and generally photodetector quantum efficiency improvement) [120,121], photocatalytic reaction improvement [122] including water splitting under solar irradiation [123], surface-enhanced Raman spectroscopy enhancement [124,125], fluorescence enhancement [126], optical trapping and optical tweezers [127], including optical and optofluidic particle manipulation [128], extraordinary optical transmission structures [129], etc.

Further uses include fabrication of ultrafast and ultrasensitive thermal detectors [130], SPASERs (surface plasmon amplification by stimulated emission of radiation), plasmonic nanolaser arrays [131], plasmonic circuits [132], plasmonic-based nanoelectronic devices [133], ultrafast resonators tunable in real time, including optoplasmonic whispering-gallery-mode microcavities [134], security and anti-counterfeit solutions [126], photodegradation of molecules detection [135], stretchable plasmonics (soft plasmonics or mechanoplasmonics) [136] and many more. Some applications of nanomembrane plasmonics are closely related with the use of plasmonic metasurfaces and nanoantennas. These will be considered in more detail in the following sections.

## 5. Membrane Metamaterials

### 5.1. Introductory Remarks and Basic Concepts

Most generally, metamaterials can be defined as composite structures with an effective response not readily met in nature, and often solely found in artificial structures. Electromagnetic metamaterials were the firstly discovered kind of such structures, and among them the materials with negative values of effective refractive index [137]. Optical metamaterials are limited to the optical range of the electromagnetic spectrum, while plasmonic metamaterials include plasmonic building blocks. A joint property of practically all metamaterials is that their building blocks have subwavelength dimensions, contrary to the photonic crystals with their mesoscopic dimensions.

Optical (and generally electromagnetic) metamaterials typically include scatterers of two kinds, one of which separately defines effective electrical behavior by determining the effective relative dielectric permittivity ε, while the other independently defines magnetic behavior by scatterers determining the effective relative magnetic permeability μ. Since each one of these parameters can be independently tailored, the desired values of ε and μ can be chosen. As mentioned in Section 2, the final electromagnetic behavior (effective refractive index) in a given spectral range will be defined by each of those parameters according to the definition of refractive index *n* stemming directly from Maxwell’s equations as $n=\sqrt{\mu \;\varepsilon }$.

Since the subwavelength scatterers can furnish ε > 0, ε < 0 or ε ≈ 0 and independently of that μ > 0, μ < 0 or μ ≈ 0, this means that several combinations are possible. Table 2 summarizes the possible cases, including the situations where ε >> 0, and represents a generalization of the information presented in Figure 1 of the reference [138].

A notable and probably the most popular example are materials with negative effective refractive index [139]. If both ε and μ are negative independently on each other in the wavelength range of interest, then for their nanocomposite we may write μ = |μ| exp (*i*μ) and in an analogous manner ε = |ε| exp (*i*ε). Thus

(4) $$n=\sqrt{\left|\mu |\;|\varepsilon |\;\exp(2i\pi \right)}=\sqrt{|\mu |\;|\varepsilon |\;}\sqrt{\exp(2i\pi )}=\sqrt{|\mu |\;|\varepsilon |\;}$$

which means that the refractive index of a nanocomposite with simultaneously negative μ and ε has to be negative. At the same time, such a composite must be dispersive and lossy in order to preserve causality principle. A more strict consideration from the point of view of causality principle may be found, e.g., in [140]. The quoted paper also tackles the question in which case it is permissible to describe a composite of two materials with independently built ε < 0 and μ < 0 components as a medium with negative effective index.

Metamaterials are designer materials, which means that their effective permittivity and permeability are tailored by design. Thus, in principle, their designer can engineer nanocomposites with any value of ε and μ (as shown in Table 2) at any desired wavelength. Maxwell’s electromagnetic equations are scale-invariant to allow one to design one’s own metamaterial for any targeted wavelength by scaling the geometrical dimensions up or down, so there are electromagnetic metamaterials for radio-waves, microwaves, millimeter waves, infrared, optical, etc. This does not stop at electromagnetics, and metamaterials have been successfully designed to deal with, e.g., sound waves (acoustic metamaterials) [141] or heat conduction (thermal metamaterials) [142].

### 5.2. Metasurfaces

Metasurfaces can be defined as planar metamaterials with subwavelength thickness that retain all wavefront tailoring functionalities of conventional metamaterials [143]. Thus, the optical metasurfaces make an almost perfect match with biomimetic nanomembranes, the latter being probably the most convenient platform for them. The compatibility of metasurfaces with planar technologies means facilitated fabrication and integration of electromagnetic scatterers (protrusions or pits/apertures) with nanomembranes and easier control over the structural and material parameters compared to the painstaking methods that are experienced with when attempting to make 3D structures. This, alongside an almost limitless versatility of the uses of metasurfaces, has been keeping this subject a hot topic of research since their inception.

The subwavelength scatterers on the metasurface can be denoted as “meta-atoms” [144], since in electromagnetic sense they actually perform a function similar to regular atoms in conventional materials. Being much smaller than the optical wavelength, they generate effective electromagnetic response through the effective parameters ε, μ, thus defining the effective refractive index *n* through a mechanism very similar to that in natural materials.

Since we are not bound by natural crystal properties, the scatterers can be fabricated in various sizes on the same surface. They can also assume different shapes on the same metasurface.

As far as the spatial layouts of electromagnetic scatterers are concerned, there are many different possibilities as well. Figure 1 shows the fundamental types of periodic 2D lattices in which meta-atoms can be organized within a metasurface. All of these are met in structures commonly found in nature.

Besides the conventional layouts of scatterers within a metasurface, it is equally possible to organize them in other types of lattices. Figure 4 shows two such unconventional structures. In reality, there is an almost limitless number of such layouts that can assume quasiperiodic or aperiodic form. They can also be fully randomly scattered over the surface. The most general case is a metasurface with a random layout of meta-atoms, where both shapes and sizes of scatterers vary.

The fact that meta-atoms can have different shapes and forms may actually introduce deeply subwavelength electromagnetic field “hotspots” within the meta-atom itself, causing the effective medium approximation to break down [145]. In this way they may cause novel phenomena, based on nonlocality effects.

**Figure 9:**
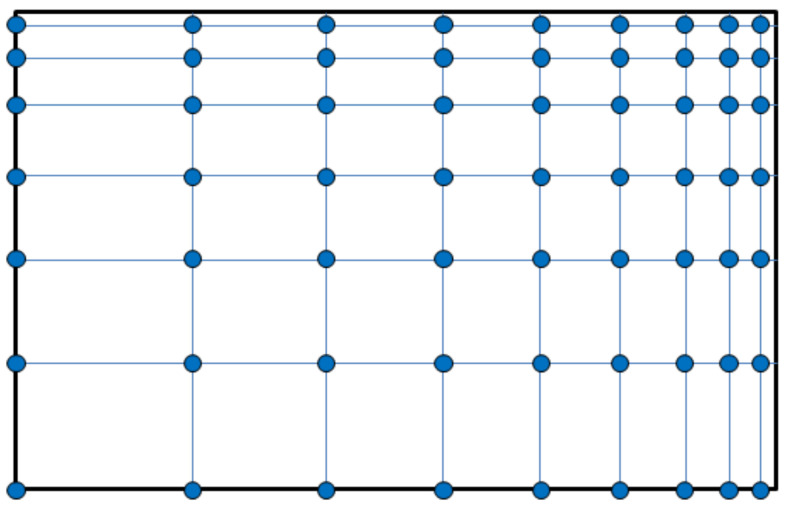
An example of a simple gradient metasurface; the structure is graded along both in-plane axes; blue circles represent meta-atoms, while blue lines denote the cells of the gradient subwavelength lattice.

Since the arrangement of meta-atoms does not have to obey the rules of conventional crystallography, metasurfaces can also have spatial gradient. The gradient metasurfaces [146] cause spatial variations of amplitude, phase, and polarization of the involved electromagnetic fields [147]. A simple example of a 2D gradient metasurface is shown in Figure 9, where there is grading of the distance between meta-atoms along both of the Cartesian axes. In [146], the authors Ding et al. classified metasurfaces to geometric, Huygens-type, reflective, electric-dipole response-based, electric-magnetic dipole response-based, magnetoelectric coupling-based, and metal-backed (reflectarrays).

Biomimetic nanomembranes are a convenient platform to relatively easily fabricate another type of metasurfaces with aperture arrays called the fishnet structures. The fishnet metasurfaces can be either single-layer or multilayer. The single-layer fishnets are actually the type of structures used to the first experimental demonstration of the extraordinary transmission effect [104]. The most often utilized multilayer fishnets are 3-layer structures of metal-dielectric-metal type, with a 2D array of holes (most often being circular or rectangular) passing throughout the multilayer. These are the metamaterial structures that proved themselves convenient for obtaining a negative effective refractive index in the optical wavelength range [148,149]. The dimensions of the apertures, as well as the total thickness of the multilayer, are both subwavelength. Multilayer fishnets are also met in literature [150].

### 5.3. Low-Loss Metasurfaces

Decreasing absorption losses in metamaterials is a subject of prime importance for a majority of metamaterial applications. Since a majority of metamaterials are dispersive resonant structures, their absorption losses will be very high, which effectively hinders a practical use of many extraordinary effects achieved in them.

Absorption losses in metamaterials generally can be decreased in several ways. One of the methods is the fabrication of metal-dielectric metasurfaces with minimized metal volume fraction. Such structures actually represent a generalization of the almost 70 years old concept of artificial dielectrics [151]. With a decrease of the metal volume fraction, a lower amount of electromagnetic energy propagates through the lossy conductor. There is a trade-off, however, because a larger percentage of lossless dielectric will mean that the field localization will be proportionally smaller.

Another method is the use of alternative free-electron conductors with decreased losses compared to customarily used good metals. These include optically transparent, electrically conductive oxides (TCO), for instance ITO–indium-tin-oxide; GZO–gallium-zinc-oxide; AZO–aluminum-zinc-oxide. Graphene and other 2D materials are also convenient. One can also use phase change materials (e.g., transition metal oxides or chalcogenide glasses) as constitutive materials of metamaterials [93]. These materials reversibly change their state from crystalline to amorphous, while their optical properties vastly vary in the process. These changes are rapid, and the structure switching can be made externally by optical or electrical excitation. The multifunctionality of phase change materials makes them convenient for nonvolatile memory storage applications, but also for a number of nanophotonic applications [152]. A treatise dedicated to alternative and lower loss plasmonic materials can be found in [94].

Absorption losses in metamaterials can be avoided by utilizing pure dielectrics with high refractive index and high anisotropy [153,154]. No metals or conductors with free electron plasma and negative permittivity are used. Orthogonal electric and magnetic dipoles of the dielectric medium are applied instead, together with a vast number of higher order multipole resonances. Mie resonant theory is used to calculate the properties of such structures. The shape, the disposition, and the spatial layout of subwavelength dielectric scatterers all determine the behavior of an all-dielectric metamaterial. Obviously, these effective materials are not plasmonic ones, but they do belong to metamaterials since they exhibit analogous traits.

A further method to be used to minimize absorption losses is to use conventional epsilon negative-epsilon positive structuring, but in this case, the frequency dispersion itself is to be optimized by design to achieve lower losses. A famous example of this is hyperbolic metamaterials and metasurfaces, whose anisotropic optical parameters can be tailored to strongly suppress losses.

Finally, nanomembranes as a building block for plasmonics have inherently lower, albeit inherently anisotropic, losses than bulk plasmonics because of their minuscule thickness that ensures a vastly shorter optical path, and thus an absorption decrease in the direction perpendicular to the nanomembrane surface.

### 5.4. Hyperbolic Metasurfaces

Hyperbolic metamaterials [155,156,157] exhibit extreme optical anisotropy and the signs of the components of their dielectric permittivity tensor in two orthogonal directions are opposite. Their isofrequency surfaces for extraordinary waves in momentum space have the form of a hyperboloid (hence the name). Depending on the value of permittivity, these hyperboloids can be open or closed. What attracted the attention of the scientific community to hyperbolic metamaterials is the fact that their absorption losses in can be strongly reduced by proper design.

**Figure 10:**
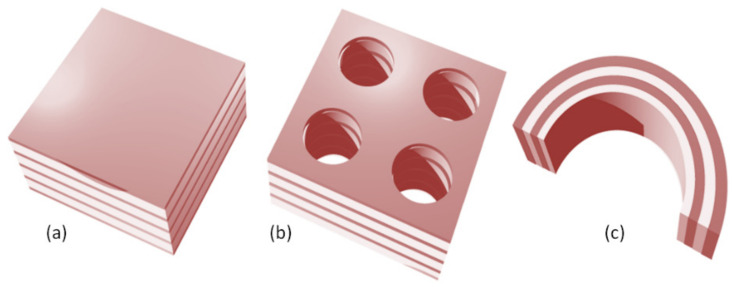
Some examples of hyperbolic metasurfaces. (**a**) simple metal-dielectric multilayer; (**b**) multilayer plasmonic fishnet; (**c**) sculpted metal-dielectric multilayer (can perform a function of a cylindrical hyperlens).

Hyperbolic metasurfaces [158,159] are actually hyperbolic materials with subwavelength thickness. A few examples of 1D hyperbolic metasurfaces are demonstrated in Figure 10a, showing a metal-dielectric (generally epsilon negative–epsilon positive) planar ultrathin film. Figure 10b shows the same film, but with apertures which make it a fishnet metasurface. Figure 10c shows the metal-dielectric multilayer sculpted in cylindrical shape which is applicable as a hyperlens.

**Figure 11:**
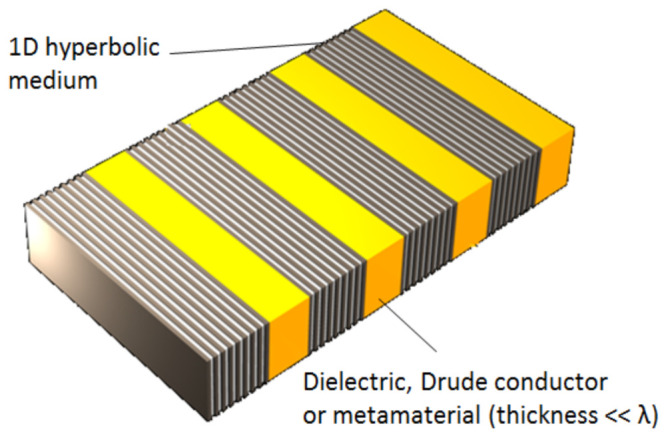
Hypercrystal metasurface containing hyperbolic metamaterial layers.

Figure 11 shows a photonic hypercrystal metasurface that can be formed by alternating layers of hyperbolic material and any other optical material (it may be dielectric, conductor with free electron plasma or metamaterial). A photonic hypercrystal [160] combines features of conventional photonic crystals with those of hyperbolic metamaterials. The surface of a hypercrystal supports different kinds of electromagnetic surface modes and states, which makes the structure extremely sensitive to even minuscule amounts of any material adsorbing to its surface. This points out towards the use of photonic hypercrystals in ultrasensitive chemical and biological sensing.

### 5.5. Applications of Metasurfaces

Plasmonic metasurfaces are utilized to fabricate emitters with controllable color, including LEDs, various sensors including chemical, biological and mechanical ones, and different biomedical devices based on nanotechnology. Their uses range from passive structures such as ultrathin metalenses, color filters, wide range absorbers, different novel light waveguides, electromagnetic switches including those for terahertz range, to active devices like various lasers.

A holy grail of the metasurfaces research is the possibility of using them as building blocks for all-optical or hybrid optical/electronic integrated circuits that would exhibit both the packaging density and the complexity of the modern integrated circuits. The concept was proposed by Ozbay [12]. The extreme field localizations obtained by the use of arbitrary field distribution tailoring enabled by metasurfaces are crucial for that purpose. Some recent works are aimed in this direction. The paper [161] considers the use of graphene-based metasurfaces to tailor the relative phase difference of two input signals and achieve their constructive or destructive interference. In this way, all-optical logic gates of AND, OR, and XOR type were achieved. The paper by Liu et al. [162] reported programmable all-optical diffractive deep neural networks based on a digital-coding metasurface array with a multilayer structure. Each meta-atom of this structure is integrated with two amplifier chips and performs the role of an active artificial neuron. The authors call their device a programmable artificial intelligence machine.

Metasurfaces enable obtaining structural colors by their ultrathin flat structures through the use of planar technologies. A large part of current research on metasurfaces is dedicated to the novel generation of color displays. In their 2020 report in *Science*, Joo et al. [163] presented OLED (organic light-emitting diode) displays that are metasurface-driven and reach resolutions beyond 10,000 pixels per inch. These displays were intended for next-generation near-eye microdisplays. Another direction of metasurface-based displays are the groundbreaking holographic ones [164,165].

A metasurface camera was described and fabricated by Arbabi et al. [166]. It operates in near diffraction-limited mode. The camera monolithically integrates a metasurface lens doublet corrected for monochromatic aberrations with an image sensor. More recent achievements in this field include high-sensitivity polarization imaging camera based on a compound-eye metasurface biomimetic design [167] and intelligent metasurface camera based on programmable metasurfaces and using machine learning [168].

Besides using the structural colors of metasurfaces for displays and cameras, another application has been proposed: the generation of photorealistic full-color still images. A photorealistic, brightness-tunable nanopainting with full-color gamut has been produced using a low-loss TiO_2_ metasurface based on spatially varying nanopillars, fully mimicking the vividness, texture, and colors of an original oil-painting [169].

Regarding the use of holographic metasurfaces, other fields of use unrelated either to displays or to images and imaging generally are researched. One of them is the fabrication of optical large-capacity data storage with extreme data densities [170].

The paper [171] considered the concept of the Huygens’ metasurfaces. These are freestanding nanomembrane structures made of high refractive index dielectric. The electromagnetic behavior of Huygens’ metasurfaces is determined by Mie resonance (both electric and magnetic) within the dielectric material. This means that Huygens’ metasurfaces actually use a subwavelength layer of orthogonal electric and magnetic dipoles. These dipoles play the role of light sources based on Huygens’ principle and are excited by the incident beam. Due to such nature, they offer an extremely high level of beamforming control. Among other thigs, they ensure perfect refraction (with zero reflection), arbitrary beam forming and perfect anomalous reflection.

The paper [171] presents a Huygens’ metasurface-based design of a beam deflector, a metalens, and a metasurface axicon [172] (lens with a conical surface, used to transform Gaussian beam into a Bessel-like one with an annular far field beam distribution, which is utilized in optical trapping). Huygens’ metasurfaces have also been proposed for the use in antenna applications [173].

## 6. Planar Nanoantennas on Nanomembranes

### 6.1. Introductory Remarks

Nanoantennas [174] represent antennas with dimensions in the nanoscale range, dedicated to working with optical radiation (including infrared) [175,176]. Because of the minuscule amounts of power related to their individual elements, they are usually fabricated in the form of arrays. Nanoantenna arrays can be regarded as special kind of metasurfaces [177], where each separate subwavelength scatterer (meta-atom) functions as a single nanoantenna for the optical range.

The nanoantenna elements can be made of plasmonic conductors, semiconductors, or they can be all-dielectric. Two-dimensional materials like graphene are also used. The nanoantenna elements will usually have low absorption losses, because even in the case of the metallic ones, they are ultrathin by definition, so the optical path will be too short to incur significant losses [177]. Their directionality will be either a consequence of their optical resonance properties or their various resonance modes interfering among themselves. Obviously, their operating frequencies will be of the order of hundreds of terahertz.

**Figure 12:**
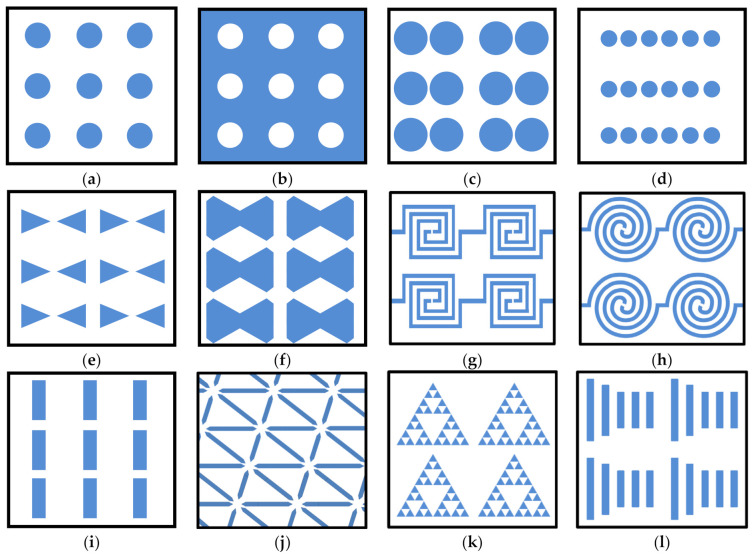
(**a**) Low-height conductive cylindrical scatterers acting as an array of electromagnetic dipoles on dielectric nanomembrane surface; (**b**) complementary structure to (**a**) with conductive surface and apertures (dielectric), designed according to Babinet principle; (**c**) nanodimers on nanomembrane; (**d**) nanodot array (nanoparticle chain); (**e**) bowtie nanoantennas array; (**f**) diabolo array; (**g**) square spiral nanoantennas array; (**h**) round spiral nanoantennas array; (**i**) end-to-end two-wire array; (**j**) hexagonal lattice array; (**k**) Sierpinski fractal nanoantenna array; (**l**) Yagi-Uda array.

To be used in collecting optical energy, nanoantennas (which inherently produce AC signals) have to be combined into circuits with fast enough rectifier elements (ultrafast diodes) to obtain a useful DC signal. These devices are known as optical rectennas (rectifying antennas). In the electromagnetic sense, optical rectennas are mostly equivalent to those already used at longer wavelengths, microwave for instance (with a few distinctions, for instance skin effect is much more pronounced at optical frequencies, and, on the other side, generalized Ohmic law has to be used). However, their practical fabrication and implementation are vastly more challenging due to the need for extremely fast diodes unhampered by parasitic capacitance (for optical rectennas its value has to be up to a few attofarads). This is the reason why in the current optical rectennas one uses metal-insulator-metal (MIM) tunneling diodes, which, contrary to the conventional diodes, are not affected by the detrimental influence of parasitic capacitances [178,179]. Figure 12 shows some selected types of nanoantennas. It presents (a) conductive cylindrical scatterer arrays [180]; (b) nanohole array in conductive surface (optical aperture nanoantennas) [181], (c) metallic nanodimers [182]; (d) nanodot array (nanoparticle chain) [183]; (e) bowtie nanoantennas [184]; (f) diabolo array [185]; (g) square spiral nanoantennas [186]; (h) round spiral nanoantennas [187]; (i) end-to-end two-wire array [188]; (j) hexagonal lattice array [189]; (k) Sierpinski fractal nanoantenna [190]; (l) Yagi-Uda array [191]. Besides the geometries shown in Figure 12, there are many other nanoantenna designs (e.g., bull’s eye, triangular lattice, V-shaped, logarithmic, Archimedean and Euler spirals, oligomer nanoantennas, crossed bowties, multiparticle common-gap antennas, etc.), not shown here.

Nanoantennas, due to their resonant response, usually have very narrow bandwidth of the order of few tens on nanometers (e.g., [192,193]), although “wideband” nanoantennas were reported with FWHM (Full Width Half Minimum) between 500 nm and 900 nm [194]. Ni et al. fabricated wideband nanoantennas for the 1 μm to 1.7 μm range [195]. Ren et al. reported in 2022 nanoantennas for the infrared range for what they call ultra-broadband region from 6 μm to 9 μm [196]. Obviously, the exact spectral ranges will depend on the particular materials and geometries used.

### 6.2. Some Applications

An in-depth review of directional nanoantennas was written by Li et al. [197]. Among other things, the team analyzed advantages and deficiencies of a large number of different types of directive nanoantennas in a systematic manner. They also considered the main problems to be solved and offered an outlook to the field.

A large number of useful functionalities are obtained through the use of nanoantennas. Sortino et al. [198] fabricated cylindrical nano-antennas in gallium phosphide (regarded as high-refractive-index dielectric with low absorption loss). They coupled them to atomically thin two-dimensional semiconducting transition metal dichalcogenides (more particularly, monolayers and bilayers of WSe_2_). The consequences of that coupling were a photoluminescence enhancement in excess of four orders of magnitude and Raman scattering signal enlargement exceeding three orders of magnitude.

Barreda et al. performed theoretical and numerical intercomparison of different nanoantennas aimed to enhance the spontaneous emission of quantum dot single photon emitters [199]. They considered different cylindrical nanoantennas made of silicon, gold and core–shells with a silicon core and gold shell. The result was that gold two-cylinder dimers ensured a high Purcell factor, while quadrupolar electric resonance in the gold dimer ensured necessary directionality.

An important field of nanoantennas is single-molecule biological sensing [181]. In the quoted paper the aperture-based nanoantenna arrays were used (Figure 12b).

## 7. Nanomembranes in General Photonics

### 7.1. Introductory Remarks

Besides the above considered classes of nanomembrane-based nanophotonic structures that all belong to either mesoscopic or subwavelength scatterer-based nanocomposites, which indeed appear vastly prevalent in the available literature [200], there are many examples that cannot be included in any of them. A lot of them use at least one of the additional features of nanomembranes unrelated to nano-optical functionalities, some of them even more than one (e.g., simultaneously transferability and tailorability through strain engineering). In some applications non-functionalized flat nanomembranes without scatterers are used, or plain nanomembranes with surface sculpting in rolled-up or origami-like fashion are utilized. For some of these cases nanomembranes only serve as supporting platforms for functional nanostructures and are unrelated to plasmonics, PBG, metamaterials, or nanoantennas. Nevertheless, they belong to the field of nano-optics in that the structures, devices, and systems basing their functions on them interact with light within subwavelength areas or volumes.

### 7.2. Applications

Next, we list some recent advancements, including single-layer nanomembranes functionalized by quantum dots, nanomembranes as the principal building blocks of resonant cavity-enhanced photodetectors where the membrane is built of the photosensitive active material of the photodetector, etc.

Regarding the use of photonic nanomembranes in photonic structures not belonging to any of the previously described classes, Aouassa et al. utilized transferable nanomembranes to fabricate solar cells [48]. The team functionalized 30 nm thin silicon nanomembranes by growing InAs/GaAs quantum dots directly on their surface. The size and bandgap width of the quantum dots were both tailored by nanomembrane strain engineering. Chen et al. fabricated resonant-cavity-enhanced photodetectors on transferable single-layer GeSn nanomembrane for the optical communications at an operating wavelength of 2 μm [47]. The thickness they used was higher than usual for nanomembranes and actually belonged to the submicrometer range, with the values of 630 nm and 918 nm. They obtain responsivities of about 0.5 A/W, more than two orders of magnitude higher than in previously reported GeSn photodetectors of the equivalent kind.

Among the advanced fields of immediate practical interest are hybrid or all-optical photonic circuits, including the programmable ones [201]. These photonic circuits include integrated interferometers (e.g., Mach–Zehnder interferometer) with multiple ports and meshes of optical waveguides. Tunable directional couplers can be also used to the same purpose. Further information about programmable photonic circuits that can benefit from ultrathin film nanophotonics can be found in [202]. Information regarding nanophotonic structures in lithium niobate thin film integrated electro-optic modulators and their scalability are considered in [203].

Another area of interest is engineering of far-field thermal radiation using general nanophotonics structures [204], for instance atomically thin 2D structures such as molybdenum sulfide for tailoring the spectral dispersion, polarization, radiation directionality, and the temporal response of thermal emitters. Microresonator-based solitons for optical communications that ensure massively parallel function are considered in [205].

Nanomembrane platforms are also of interest for nonlinear optics [65] (nanomembrane origami), [206]. Strong coupling tailors the energy states of molecules, with applications in, e.g., Bose–Einstein condensation, polariton lasing, superfluidity, Raman scattering, but also quantum information processing, etc.

Another area for which nanomembranes exhibit importance is quantum optics [207,208] (used to combine classical electrodynamics with quantum mechanics, and resulting in applications like generating repulsive forces, creating photons from vacuum, and generally tailoring material functionality). The latter points out to the use of quantum effects in realization of quantum photonic integrated circuits [209].

Besides, in the choice application fields outlined above, nanomembrane platforms are used in producing different passive [210] and active nanophotonic components [211]. Obviously, their applications cover a vastly wider field, but covering it at least partly by far exceeds the scope of this study.

## 8. Conclusions

In this review, we made an attempt to systematize some properties and applications of the new nanosystem building block and the biomimetic nanomembrane, within the context of the wide field of nano-optics/nanophotonics. For this purpose, we investigated planar versions of some of the most often used periodic, quasiperiodic, or aperiodic nanophotonic nanocomposites, including photonic bandgap materials, subwavelength plasmonic crystals, optical metasurfaces, and planar nanoantennas. We also overviewed some recently proposed practical applications of these concepts. Obviously, a large percentage of the applications will overlap among the quoted fields because the approaches are based on the common foundations and there are various alternative approaches to reach the same goal. Inevitably, many applications will remain uncovered by this treatise, since the field handled by it is extremely wide and it would need a much wider text to systematize such richly diversified area.

We also mention here some unconventional properties of nanomembranes not related to their optical behavior that are used to further multifunctionalize ultrathin freestanding photonic structures and devices and impart them additional useful properties. Such non-optical traits include self-reparability, transferability to arbitrary substrates, antifouling properties, extreme stretchability, flexibility and foldability, and the possibility of 3D sculpting (facile forming of curled structures, origami-inspired geometries, etc.)

Using biomimetic nanomembranes to tailor nanoscale propagating and evanescent electromagnetic fields has already opened many new research and application pathways. It is continuing to expand the horizons within the area with an ever-increasing speed. Shortly, the use of biomimetic nanomembranes has offered nanophotonics researchers an extensive and extremely powerful design toolbox. Nanomembranes in nano-optics and nanophotonics can be thus described as real metastructures, since they do go beyond the natural structures and open pathways to a wide variety of novel functionalities.

A possible direction of the future work, besides continuing the already extremely variegated research in the related fields, would be merging biological efficiency and elegance with nanophotonics, and ensuring a further pathway towards biologically driven intelligent applications. Another important branch are hybrid structures, integrating different physical mechanisms to achieve structures and devices going beyond the state of the art, e.g., the combination of nanophotonics with spatiotemporal modulation, their enrichment with acoustic, thermal, and electrical multifunctionalities, and generally light-matter interactions that have been less researched until now.

We hope that this review of some present directions of investigation of nanophotonic structures and devices with quasi-2D building blocks could prove handy to researchers in the field. At the same time, it was our intention to keep this treatise readable, accessible and of interest to general scientific community.

## Figures and Tables

**Table 1:** Classification of synthetic nanomembranes for nano-optics based on their composition.

Synthetic Nanomembranes for Nano-Optics	Types of Nanomembranes
Inorganic	Pure metal (e.g., gold, chromium) [23] Metal composites (mixed matrix) and alloys (e.g., Cr + Si) [24]
	Diamond (nanocrystallite sheets) [25] Diamondoids (adamantane, tetramantane) [26] Diamond-like carbon (DLC) nanomembranes (hard carbon allotrope) [27] Carbon nanomembranes–cross-linked carbon precursors [28]
	Single element semiconductors (silicon, germanium) [29] Compound semiconductors [30]
	Freestanding monatomic sheets (e.g., graphene, borophene) [31] Twisted bilayers-moiré structures (e.g., graphene bilayers) [32] Freestanding inorganic monomolecular sheets (e.g., MXenes) [33]
Organic-inorganic hybrids	Interpenetrated structures (e.g., polyacrylate interpenetrated with ZrO_2_, SiO_2_) [34] Metal–organic frameworks (metal ions or ion clusters and organic molecules) [35]
Organic (polymer-based)	Single-polymer (pure)–e.g., polyester, polystyrene [36] Copolymer (2 or more different polymers blended) [37]

**Table 2:** Summary of achievable types of nanocomposites according to the values of their effective permittivity and permeability.

ε, μ	Type of Waves	Natural/Artificial	Name
ε < 0 μ > 0	Evanescent	Metals Drude-type free electron conductors	ENG (epsilon-negative)
ε > 0 μ > 0	Propagating	Lossless dielectrics Low-loss semiconductors	PRM (positive refractive index materials)
ε < 0 μ < 0	Propagating	Artificial	DNG (NRM) (double-negative materials) (negative refractive index)
ε > 0 μ < 0	Evanescent	frequencies below GHz: gyrotropic magnetics optical frequencies: artificial	MNG (magnetic permeability negative)
ε ≈ 0 μ ≈ 1	Evanescent	Artificial	ENZ (near-zero permittivity) (near-zero refractive index)
ε >> 0 μ > 0	Propagating	Artificial	EVL (permittivity very large) (refractive index very large)
